# Exploring Acceptability of Employment Interventions to Support People Living With Cancer: Qualitative Study of Cancer Survivors, Health Care Providers, and Employers

**DOI:** 10.2196/47263

**Published:** 2023-06-26

**Authors:** Rachel C Forcino, Sivan Rotenberg, Kali J Morrissette, Cassandra M Godzik, Jonathan D Lichtenstein, Jenna E Schiffelbein, Courtney J Stevens, Vidya Sundar, Debra L Brucker, Deirdre Connolly, Julie Keysor, Kathleen Doyle Lyons

**Affiliations:** 1 The Dartmouth Institute for Health Policy and Clinical Practice Geisel School of Medicine Dartmouth College Lebanon, NH United States; 2 Department of Psychiatry Dartmouth-Hitchcock Medical Center Lebanon, NH United States; 3 Dartmouth Cancer Center Geisel School of Medicine Lebanon, NH United States; 4 Occupational Therapy Department University of New Hampshire Durham, NH United States; 5 Institute on Disability University of New Hampshire Durham, NH United States; 6 Occupational Therapy Department Trinity College Dublin Ireland; 7 Physical Therapy Department Massachusetts General Hospital Institute of Health Professions Boston, NH United States; 8 Occupational Therapy Department Massachusetts General Hospital Institute of Health Professions Boston, MA United States

**Keywords:** cancer, employment, intervention development, intervention, people living with cancer, cancer survivor, health care provider

## Abstract

**Background:**

Employment contributes to cancer survivors’ quality of life, but this population faces a variety of challenges when working during and after treatment. Factors associated with work outcomes among cancer survivors include disease and treatment status, work environment, and social support. While effective employment interventions have been developed in other clinical contexts, existing interventions have demonstrated inconsistent effectiveness in supporting cancer survivors at work. We conducted this study as a preliminary step toward program development for employment support among survivors at a rural comprehensive cancer center.

**Objective:**

We aimed (1) to identify supports and resources that stakeholders (cancer survivors, health care providers, and employers) suggest may help cancer survivors to maintain employment and (2) to describe stakeholders’ views on the advantages and disadvantages of intervention delivery models that incorporate those supports and resources.

**Methods:**

We conducted a descriptive study collecting qualitative data from individual interviews and focus groups. Participants included adult cancer survivors, health care providers, and employers living or working in the Vermont–New Hampshire catchment area of the Dartmouth Cancer Center in Lebanon, New Hampshire. We grouped interview participants’ recommended supports and resources into 4 intervention delivery models, which ranged on a continuum from less to more intensive to deliver. We then asked focus group participants to discuss the advantages and disadvantages of each of the 4 delivery models.

**Results:**

Interview participants (n=45) included 23 cancer survivors, 17 health care providers, and 5 employers. Focus group participants (n=12) included 6 cancer survivors, 4 health care providers, and 2 employers. The four delivery models were (1) provision of educational materials, (2) individual consultation with cancer survivors, (3) joint consultation with both cancer survivors and their employers, and (4) peer support or advisory groups. Each participant type acknowledged the value of providing educational materials, which could be crafted to improve accommodation-related interactions between survivors and employers. Participants saw usefulness in individual consultation but expressed concern about the costs of program delivery and potential mismatches between consultant recommendations and the limits of what employers can provide. For joint consultation, employers liked being part of the solution and the possibility of enhanced communication. Potential drawbacks included additional logistical burden and its perceived generalizability to all types of workers and workplaces. Survivors and health care providers viewed the efficiency and potency of peer support as benefits of a peer advisory group but acknowledged the sensitivity of financial topics as a possible disadvantage of addressing work challenges in a group setting.

**Conclusions:**

The 3 participant groups identified both common and unique advantages and disadvantages of the 4 delivery models, reflecting varied barriers and facilitators to their potential implementation in practice. Theory-driven strategies to address implementation barriers should play a central role in further intervention development.

## Introduction

Employment contributes to cancer survivors’ quality of life, both through the contribution of income to well-being [1,2] and through fostering purpose and sense of self [3]. However, most cancer survivors working at the time of diagnosis make cancer-related adjustments at work, including taking extended time off, working part time, or declining promotion [4]. These adjustments have long-term impacts; cancer survivors are also more likely than peers without cancer to be unemployed, even up to 15 years after cancer diagnosis [5]. Support is therefore needed to help cancer survivors maintain employment.

Prior research has identified seven broad factors associated with work outcomes among cancer survivors: (1) cancer survivor social and demographic characteristics; (2) health and well-being, including medical, behavioral, and social health; (3) symptoms, including fatigue, pain, and cognitive issues; (4) function, including physical, cognitive, emotional, and interpersonal aspects; (5) work demands, including physical, cognitive, emotional, and interpersonal demands; (6) work environment, including flexibility, support, climate, and job stress; and (7) policies, procedures, and economic factors in organizational, legal, and financial contexts [6]. In our rural setting, three specific work-related issues reported by cancer survivors, clinicians, and employers include (1) an onus on the cancer survivor to identify and articulate barriers, (2) time away from work as the main solution to alleviating work issues, and (3) a lack of information available to cancer survivors, clinicians, and employers about optimizing cancer survivors’ employment situations [7].

At least 3 systematic reviews, including a Cochrane review [8], have been conducted in the past 7 years to synthesize the evidence base regarding employment support for cancer survivors [9,10]. Interventions tested in the cancer context include psychoeducation in individual and group formats, medical or procedural interventions, physical activity training, vocational support, and multidisciplinary interventions combining 2 or more of these components [8]. The 2015 Cochrane review concluded that multidisciplinary interventions, which included physical, psychoeducational, and vocational content had the greatest potential to foster higher employment rates for cancer survivors. However, the effect was small, that is, the relative risk of having a higher employment rate compared to usual care when assessed up to 12 months post cancer diagnosis was 1.03-1.16 [8].

More recent reviews affirm that finding and reveal other insights. Algeo et al [9] noted that most interventions measuring employment outcomes focus on reducing physical and psychological impairments, while only a minority of interventions include vocational content. This finding was echoed in a scoping review of work-related interventions for breast cancer survivors, where only 38% included vocational content [11]. Additionally, most of the interventions reviewed did not report the use of a theoretical framework in their development [9,11]. Collectively, these reviews suggest that the field does not yet have an evidence-based approach to help cancer survivors to meet their employment goals and priorities. Incorporating targeted vocational supports may improve outcomes; however, it is not yet clear how best to deliver this type of content in the context of oncology care. The challenges of delivering psychosocial oncology care are compounded in rural settings, where clinical workforce shortages and social constraints such as the availability of local support and lengthy travel times to clinical settings can limit access [12]. Thus, behavioral intervention development research is needed to address this gap.

Stakeholder engagement is crucial when designing a new intervention or planning to implement an existing intervention in a new setting [13,14]. Our team, situated in the health care system, engaged cancer survivors and health care providers from our local setting, as we sought the perspectives of those experiencing cancer-related problems at work and those treating them. We also prioritized employer perspectives to identify their available resources and needs in supporting employees with cancer. Accordingly, we contacted 3 types of participants whose perspectives were essential when trying to identify feasible, effective ways to help cancer survivors maintain employment during and after treatment: cancer survivors, health care providers, and employers. We engaged participants in a descriptive study, using individual interviews followed by focus groups. The individual interviews generated data regarding employment challenges [7], and resources and supports that may address those challenges. These data helped us identify the types of interventions that could be developed, tailored, and deployed to support cancer survivors in maintaining employment. The focus groups provided an opportunity to present those potential interventions and identify the advantages and disadvantages of each option, generating data regarding what strategies may be needed to implement each intervention.

This paper summarizes those data to answer two questions: (1) What supports and resources do stakeholders think might help cancer survivors to maintain employment? (2) What are the advantages and disadvantages of implementing interventions that incorporate those supports and resources?

## Methods

### Design Overview and Setting

The study was the first step in the process of developing and implementing employment support services for cancer survivors served by a National Cancer Institute–designated Comprehensive Cancer Center located in a rural region of northern New England. This descriptive study collected qualitative data from individual interviews and focus groups involving cancer survivors, health care providers, and employers. The study had three steps: (1) we conducted individual semistructured interviews with a convenience sample of cancer survivors (target n=15), health care providers (target n=15), and employers (target n=15); (2) we reviewed interview content to summarize program recommendations; and (3) we conducted focus groups with the original participants to present options for program development (drawing on existing social and behavioral intervention delivery models) and obtain their feedback regarding the best ways to proceed in designing services.

### Ethical Considerations

The study was reviewed and approved (with exempt status) by 2 institutional review boards (Dartmouth-Hitchcock Health and Massachusetts General Brigham Health), and all participants affirmed their informed consent via signature prior to the first interview. Interview and focus group participants were each offered a US $50 honorarium. Participant data were stored securely and were only accessible by approved study team members on an as-needed basis.

### Participants

We recruited a convenience sample of participants who live and work in the 2-state catchment area of the Dartmouth Cancer Center located in Lebanon, New Hampshire. The cancer center is situated in a micropolitan area more than 100 miles from the nearest major metropolitan area. Almost half the catchment area population resides in rural areas, with 19% and 28% living in large rural and small rural communities, respectively [15,16]. The population is largely racially homogenous, with more than 94% of the population identifying as White [16]. The inclusion criteria were:

1. Cancer survivor: Any person 18 years and older, who has been diagnosed with any cancer type, is employed, but reports needing to work fewer hours or experiencing reduced productivity at work due to their health.
2. Health care provider: Any person who actively provides clinical or supportive care to oncology patients.
3. Employer: Any business owner, human resources professional, or manager who oversees employees. Because we were focused on the needs and resources within our catchment area, we excluded people who were living or working outside of New Hampshire or Vermont.

Cancer survivor and employer participants were recruited through flyers, advertisements, e-newsletters, social media posts, direct email invitations, and through outreach via organizations such as chambers of commerce and cancer control organizations. Health care provider participants were recruited through email invitations from the research team members who worked at the Dartmouth Cancer Center. At the end of each interview, the interviewer asked participants if they would share study contact information with others (eg, their employers) who might be willing to participate in the study.

### Data Collection and Management

#### Individual Interviews

We began with individual interviews because they allow for in-depth elicitation of perspectives on topics that could be sensitive (eg, social or financial challenges personally faced as a worker or employer). Interviews were conducted between June 2021 and January 2022 via Zoom telecommunications software, except for 1 participant who chose to be interviewed in person at the Dartmouth Cancer Center. The interviewer (KJM or CMG) used a semistructured interview guide customized for each type of participant (cancer survivor, health care provider, or employer). The interviewer asked participants to describe (1) the challenges they face or witness when cancer survivors work during treatment or return to work after taking time off; (2) the types of conversations each of these participants have with each other type of participant about work; (3) the resources that are available to cancer survivors and employers; and (4) any programs, resources, or information they feel we should develop and offer at our cancer center. These interviews are the data source for all of the recommendations about what types of employment support to offer that we report in this paper, which we modeled on existing behavioral interventions.

The individual interviews were professionally transcribed verbatim. The transcripts were proofread and edited for accuracy by a research assistant who listened to the recording. The transcripts were uploaded to NVivo (QSR International) for analysis.

#### Focus Groups

All interview participants later received an email invitation to attend a focus group during which the study team would present results from the individual interviews and solicit feedback regarding next steps. We chose to conduct focus groups because they allow participants to hear and respond to others’ perspectives regarding the advantages and disadvantages of different interventions and implementation strategies. We conducted separate focus groups for each type of participant because we wanted to minimize the degree to which social pressure would stifle the conversation (eg, employers not wanting to appear insensitive in the presence of cancer survivors or cancer survivors not wanting to criticize employers or health care providers).

Focus groups began with an orientation to the study and a 10-minute presentation of results from the individual interviews. Participants were then asked to react and add to the results. Next, participants were presented with 4 types of interventions that could be developed as the next step in this line of service research (described in the analysis section below). Participants were asked to identify the advantages of each type of intervention and what would make them want to recommend or use the intervention. They were then asked to identify the disadvantages of each intervention including the factors that would make them disinclined to recommend or use the intervention.

The focus groups were conducted in May 2022 and recorded via Zoom (Zoom Telecommunications). One facilitator (KDL) took notes, while another led the group discussion (RCF or CMG). Two team members (RCF and KJM) independently and sequentially listened to the recordings and edited the notes created by the cofacilitator to ensure that every opinion voiced by a participant was recorded accurately. These focus groups are the data source for all pros and cons of interventions that we report in this article.

### Analysis

**Figure 1:**
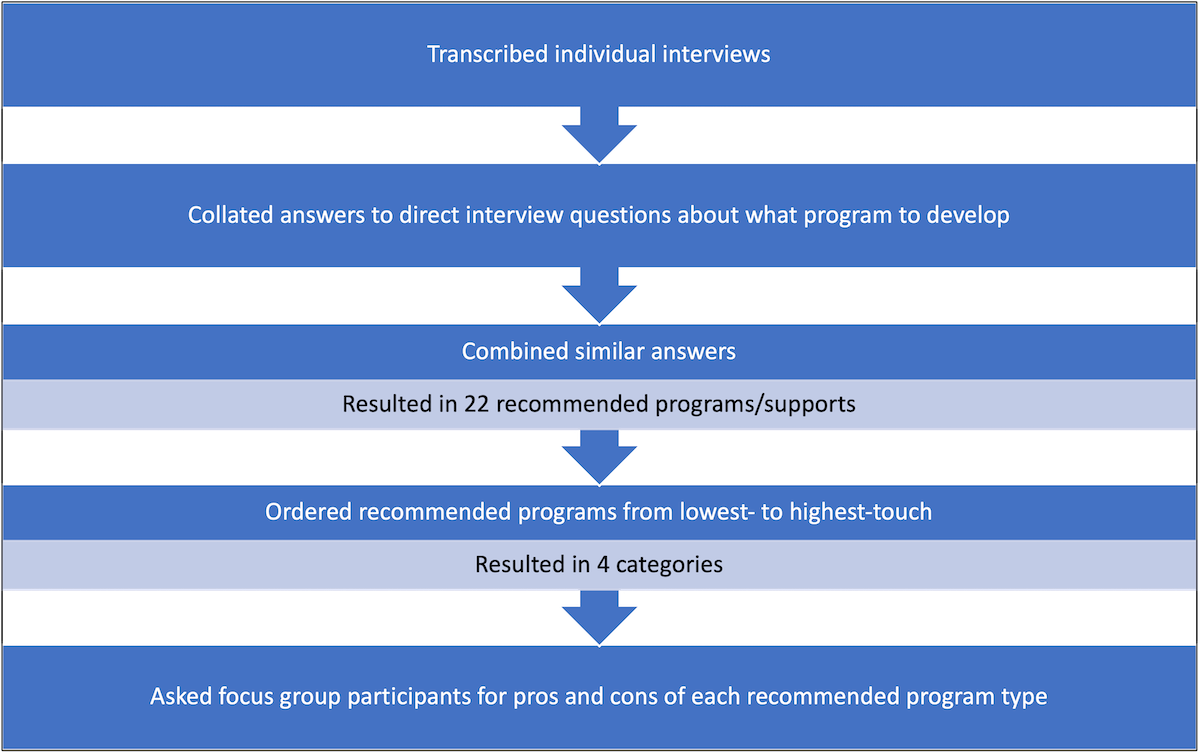
Analytic steps.

In an earlier publication using the same data set [7], we conducted a thematic analysis using direct quotations to describe overarching themes that depicted commonalities among the experiences of the participants. In contrast, our goal in this analysis was to condense and summarize the full range of perspectives and recommendations of our study’s participants. As such, we performed a content analysis to summarize our data. We used the following steps of meaning condensation and categorization that were described by Bachiochi and Weiner [17]. First, we repeatedly read the transcriptions and notes from the interviews and focus groups. Next, we iteratively developed a coding scheme to categorize the various comments made by participants in response to the open-ended questions. Finally, we made sure that each comment was captured by the coding scheme. Our application of these steps of content analysis are described below and depicted in Figure 1.

During the initial coding of the interview transcripts, described in detail in a related thematic analysis of these data [7], we created a code titled “Suggestions for what might help,” with an operational definition of “Direct advice regarding what should be included in a program designed to support employment goals of cancer survivors.” The coded text included examples of strategies and resources that participants (ie, cancer survivors, health care providers, or employers) either experienced or felt were important resources for us to develop. As we focused potential interventions on supporting survivors to remain in the workforce, we omitted suggestions focused on supporting survivors to take extended time off work, providing financial aid, and helping to file disability paperwork.

The coded text was exported to a Word (Microsoft) document. We then created a bulleted list capturing the essence of each recommendation. The list included the study identification number of the respondents who endorsed each option. Based on existing interventions that we were aware of [18-22], we then clustered items on the list into the following four categories of support: (1) education in the form of workbooks or webinars; (2) individual consultation focused on functional capacity evaluation, self-management, and empowering cancer survivors to build capacity and seek accommodations; (3) services that provide worksite evaluation and guidance to both cancer survivors and their employers; (4) employment-related peer support or advisory groups. After the categories were presented to the focus group participants, we summarized the advantages and disadvantages of developing an intervention focused on each of the 4 categories, and which type of participant identified the advantage or disadvantage. Figure 1 summarizes the analytic process.

## Results

### Participants

Forty-five people participated in semistructured individual interviews: 23 cancer survivors, 17 health care providers, and 5 employers. Fewer people participated in the focus groups: 6 cancer survivors, 4 health care providers, and 2 employers. Among the 23 cancer survivors who participated in interviews, there were 5 office or administrative support professionals, 3 primary or secondary school teachers, 2 health or social service practitioners, 2 top executives at nonprofit organizations, 2 management professionals, 2 business operations specialists, 1 farmer or rancher, 1 education administrator, 1 art and design professional, 1 recreation worker, 1 sales representative, 1 construction trades worker, and 1 truck driver. Additional participant demographics are presented in Table 1.

**Table 1:** Participant characteristics.

Characteristics		Cancer survivors		Health care providers		Employers	
		Interview sample (n=23)	Focus group sample (n=6)	Interview sample (n=17)	Focus group sample (n=4)	Interview sample (n=5)	Focus group sample (n=2)
**Gender**							
	Female	19	6	14	4	3	2
	Male	4	0	3	0	2	0
**Age**							
	Mean (SD)	55 (10.998)	50 (13.557)	51.7 (11.447)	58.3 (4.55)	51.4 (9.891)	59 (2)
	Range	28-78	27-68	27-74	55-66	34-61	57-59
**Ethnicity**							
	Hispanic or Latino	0	0	0	0	0	0
	Non-Hispanic or Latino	23	6	17	4	8	2
**Race**							
	American Indian/Alaska Native	0	0	0	0	0	0
	Asian	0	0	1	0	0	0
	African American/Black	0	0	0	0	0	0
	Native Hawaiian/Pacific Islander	0	0	0	0	0	0
	White	23	6	16	4	8	2
**Education**							
	<12 years (did not graduate)	0	0	0	0	0	0
	12 years or completed high school	1	0	0	0	1	1
	Post high school training other than college (vocational or technical)	1	1	0	0	0	0
	Some college	4	0	0	0	2	1
	College graduate	7	0	3	0	0	0
	Postgraduate	10	5	14	4	2	0
**Current employment status**							
	Full-time	14	3	12	4	7	2
	Part-time	6	2	5	0	1	0
	Retired	0	0	0	0	0	0
	Homemaker	0	0	0	0	0	0
	On long- or short-term disability	2	1	0	0	0	0
	Unemployed (looking for work)	0	0	0	0	0	0
	Unemployed (not looking for work)	0	0	0	0	0	0
	Other	1	0	0	0	0	0
**Cancer staging**							
	0	0	0	—	—	—	—
	I	4	4	—	—	—	—
	II	6	0	—	—	—	—
	III	5	1	—	—	—	—
	IV	5	0	—	—	—	—
	Unsure	3	1	—	—	—	—
**Treatment underwent**							
	Surgery only	1	1	—	—	—	—
	Chemotherapy only	2	0	—	—	—	—
	Radiation only	0	0	—	—	—	—
	Surgery and chemotherapy	5	1	—	—	—	—
	Surgery and radiation	2	0	—	—	—	—
	Surgery, chemotherapy, and radiation	13	4	—	—	—	—
**Completed treatment**							
	Yes	17	6	—	—	—	—
	No	6	0	—	—	—	—
**Occupation**							
	Physician	—	—	5	1	—	—
	Advanced practice registered nurse	—	—	4	1	—	—
	Registered nurse	—	—	2	0	—	—
	Social worker	—	—	3	0	—	—
	Occupational therapist	—	—	1	0	—	—
	Psychologist	—	—	1	1	—	—
	Dietician	—	—	1	1	—	—
**Job classification^a^**							
	Manufacturing	—	—	—	—	1	1
	Finance	—	—	—	—	1	0
	Nonprofit	—	—	—	—	1	0
	Government run (state or local)	—	—	—	—	2	1
	Health care	—	—	—	—	1	0
	Biotech or medical research	—	—	—	—	1	0
	Other	—	—	—	—	1	0
**Role^a^**							
	Owner	—	—	—	—	2	0
	Supervisor/Manager	—	—	—	—	3	0
	Human resources	—	—	—	—	3	2
	Occupational medicine	—	—	—	—	0	0
	Other	—	—	—	—	0	0
**Organization size^a^**							
	Micro (1-9 employees)	—	—	—	—	1	0
	Small (10-49 employees)	—	—	—	—	1	0
	Medium (50-249 employees)	—	—	—	—	2	1
	Large (250+ employees)	—	—	—	—	4	1
**Organization ownership^a^**							
	Locally owned	—	—	—	—	4	1
	Regional	—	—	—	—	1	0
	Part of national company	—	—	—	—	1	0
	International	—	—	—	—	2	1

^a^Three interview participants gave their perspective as both a cancer survivor and employer, thus n=8 for employers in this section.

### Education (eg, Workbooks or Webinars)

Textbox 1 contains the first category of recommendations for intervention development that involved education for cancer survivors, health care providers, and employers. This included a request for a toolkit that explains benefits and rights, examples of accommodations, and typical emotions and experiences that accompany the return to work. Notably, the list of recommendations contained several items that specifically targeted employers. For example, participants of all types felt it was important for employers to understand the diversity across types of cancers, their corresponding treatments, and how people respond to and recover from treatment. They also said it was important to illustrate how working can contribute to recovery from cancer and how workplace loyalty can be generated via support and goodwill from employers during cancer recovery.

**Potential employment-focused interventions and their advantages and disadvantages: supports related to information and education.**

**Gap in our care and support needed:**

- Screen for employment issues and refer cancer survivors to resources (*cancer survivors* and *health care providers*).
- Educate health care providers on employment resources, for example, who to refer to and for what services (*health care providers*).
- Teach employers (by video or live lecture) how to talk to people with cancer, for example, what not to say (*cancer survivors* and *employers*) and to be compassionate and show the employee you support them and have confidence in them even when they aren’t at their best (*cancer survivors*).
- Teach employers that all cancers and cancer treatments are different, that people respond differently and what types of support might be needed that some challenges are short term, and some are long term (*cancer survivors*, *health care providers*, and *employers*).
- Teach employers that work can be therapeutic and help the person recover (*cancer survivors*).
- Teach employers that having support from employers will make the employee loyal and grateful and they will want to give back (*cancer survivors*).
- Create and distribute a toolkit with information about benefits, but also about strategies to help you at work, examples of accommodations people have needed, explain what is normal and expected to struggle or feel certain ways when back at work (*cancer survivors* and *employers*).
- Employers and labor advocates create information about rights and resources (*health care providers*).

**Possible intervention: Screen for work-related distress and provide cancer survivors and employers with workbook, video, lecture, education website, and list of services and resources**

**Pros**

- Getting lots of information is helpful for some patients, especially in multiple formats (print, on the internet) (identified by *cancer survivors*, *health care providers*, and *employers*)
- Could be tailored to facilitate communication between patients and employers (identified by *cancer survivors* and *employers*)

**Cons**

- Readability and applicability across populations and professions can be an issue (identified by *health care providers*)
- Patients are overwhelmed with information at diagnosis, and it is hard to advocate for yourself anyway while you are in treatment (identified by *cancer survivors*)

In the focus groups, each type of participant acknowledged the value of providing education, and both cancer survivors and employers noted that education resources could be crafted to facilitate better interactions between survivors and employers (eg, a template for disclosure conversations). However, two important caveats were identified in the focus groups: (1) health care providers noted the challenge involved with making educational materials that can be understood by diverse populations and that apply to people with various types of jobs. (2) Cancer survivors noted that they are often overloaded with information regarding their diagnosis, and it can be challenging to read and independently apply educational information when they are undergoing treatment and not feeling well.

### Individual Consultation Focused on Self-management

Textbox 2 contains suggestions that involved individualized consultations between cancer survivors and specialists (eg, occupational therapists, social workers, and vocational rehabilitation specialists) that could evaluate and help survivors maximize work capacity, monitor progress, and identify accommodations. Other suggestions in this category indicated an interest in a program that would help cancer survivors evaluate the role of work in their lives and how they might navigate the decision to leave the workforce, change jobs, or adjust their job to better fit their priorities. The suggestions involving individual consultation and self-management approaches were all generated by cancer survivors and health care providers, which is reasonable considering employers may have little to no involvement in this type of program.

**Potential employment-focused interventions and their advantages and disadvantages: supports related to individual coaching.**

**What interviewees said was needed:**

- Provide care coordinator or nurse who can track your recovery and progress back to work, who checks in on you periodically; someone not at your workplace, maybe someone neutral but with some medical knowledge of what you have been through (*cancer survivors*).
- Functional rehabilitation services with both physical and mental components (*health care providers*).
- Need interventions that have different pathways depending on the situation (how long a person has been out of work, working part time vs out on disability) (*health care providers*).
- Intervention to help people think about the role of work in their lives given cancer diagnosis and treatment and re-prioritize if needed, make sure choices have meaning ( *cancer survivors*).
- Help people to reframe and focus on what body can do, how strong you are for making it through and shift away from negative frame of mind (*cancer survivors*).
- Shared decision-making intervention about whether to work (*health care providers*).

**Possible intervention: Individual consultation focused on self-management and empowering people to build capacity and seek accommodations**

**Pros**

- Personalized, patient-centered, and interdisciplinary (drawing on clinical team strengths) (identified by *cancer survivors* and *health care providers*)
- Provides clarity on goals and minimizes stress (identified by *cancer survivors* and *health care providers*)

**Cons**

- Potential costs to patient; challenge to fund this program (identified by *cancer survivors* and *health care providers*)
- Employer may not be able to accommodate what employment specialist recommends/counsels (identified by *cancer survivors* and *employers*)
- Possible legal issues (identified by *health care providers*)

Focus group participants appreciated the individualized nature of this type of approach and its potential to reduce stress by directly addressing cancer survivors’ issues; however, cancer survivors and health care providers had concerns about the cost of these services and how they might be funded. Employers and survivors voiced concern that the specialist would suggest strategies to the cancer survivor that would be unacceptable to the employer, and health care providers worried about liability issues that might accompany providing advice about rights and accommodations.

### Services That Provide Consultation and Guidance to Both Cancer Survivors and Their Employers

Textbox 3 contains recommendations from cancer survivors, health care providers, and employers in which a specialist would interact with both cancer survivors and employers to identify accommodations and ways to support work performance and satisfaction. This reflected a desire to improve communication and to have both parties understand and respect the other’s point of view to come to mutual solutions.

**Potential employment-focused interventions and their advantages and disadvantages: supports related to coaching that interfaces with both employee and employer.**

**What interviewees said was needed:**

- Provide a return-to-work coordinator who monitors and refers to services and interfaces with both providers and employers (*health care providers*).
- Help both parties know how to talk to each other (*employers*)—it’s not completely about the employee and not completely about the employer. Employer needs to know what employee is going through, but the employee needs to say I know this job is important, I know this is what you hired me for, I want to do x for the team/company/job (*cancer survivors*).
- Some feedback and communication procedure to know if any accommodations are working (*health care providers*).
- Offer formal occupational medicine services to patients with cancer (*health care providers*).
- Vocational rehabilitation (*cancer survivors*).
- Would be good for employers to be able to break down silos, for example, to reassign to another department if that would be a good accommodation or would be good for people looking for a career change (*employers*).
- Educate employers on the laws around accommodations and help them determine what accommodations they might be able to offer to your patient (*health care providers*).

**Possible intervention: Workplace assistance from an employment specialist who can mediate between employer and employee**

**Pros**

- Makes the employee feel supported (identified by *cancer survivors*, *health care providers*, and *employers*)
- Three-way dialogue, enhanced communication, and transparency (identified by *employers*)

**Cons**

- Needs to be positioned so it can also support self-employed patients (identified by *cancer survivors*)
- Some worksites have their own services, which employers may not allow (identified by *cancer survivors* and *health care providers*)
- Specialist may recommend things employers can’t offer (identified by *cancer survivors*)
- Unwanted attention from colleagues or special treatment (identified by *cancer survivors* and *health care providers*)
- May be better to focus on self-management and use the employer’s existing resources (identified by *health care providers*)
- Logistical hurdles, for example, extra meeting time (identified by *cancer survivors*, *health care providers*, and *employers*)

Employers voiced an appreciation of the ability of this approach to enhance communication, and they liked being part of the solution. The potential drawbacks included the hurdles of coordinating appointments and communication among more people and the ability to interface with all types of workers and workplaces. For example, cancer survivors wanted to be sure that this type of resource would be available to people who are self-employed, and both cancer survivors and health care providers worried that such a service could conflict or compete with existing services an employer might offer.

### Employment-Related Support or Peer Advisory Groups

The final recommendation came from cancer survivors who voiced an interest in a group experience—potentially peer-led—where cancer survivors could share their ideas and experiences (Textbox 4). Survivors and health care providers saw the benefits that come with group interactions in terms of the efficiency of reaching many people at one time and the efficacy of peer support. Yet, they also voiced concerns that work and the financial aspects of work can be sensitive subjects. Thus, discomfort talking about these topics with others or a distaste for group interactions might limit the accessibility of this approach. Employers also voiced a concern that group meetings could involve even more time away from work.

**Potential employment-focused interventions and their advantages and disadvantages: supports involving peers.**

**What interviewees said was needed:**

- Teach employers how to create a survivor support group at work or peer survivor advisory board to support each other (*cancer survivors*).

**Possible intervention: A support group intervention**

**Pros**

- Positive patient outcomes often come with peer sharing (identified by *cancer survivors* and *health care providers*)
- Group delivery is efficient for staffing (identified by*health care providers*)

**Cons**

- Might need to give extra time off for group meetings (identified by *employers*)
- Some patients don’t like groups and especially don’t like talking with peers about money (identified by *cancer survivors* and *health care providers*)

### Areas of Convergence and Divergence

The results demonstrate many areas of convergence, where the 3 types of participants agreed upon the needs, benefits, and drawbacks of certain approaches for supporting cancer survivors’ employment goals. For example, both health care providers and cancer survivors wanted to ensure any proposed program would meet the needs of diverse patients. Providers advocated for making sure materials were able to be read and understood by people with varying levels of literacy and survivors advocated for making sure programs met the needs of people who are self-employed.

There were some aspects in which the opinions of the 3 types of participants diverged. Health care providers pointed out some drawbacks not raised in the other focus groups. They were attuned to issues of efficiency (ie, that groups could allow them to help more patients in the same amount of time), liability (ie, a fear of providing inaccurate information), and scalability (ie, advocating for a self-management model that would fit within the realm of health care). The cancer survivors were the only group to voice the concern that, while education is important, survivors often experience information overload during diagnosis and treatment; therefore, information alone may not be helpful to survivors. Cancer survivors also worried about receiving recommendations from an individual consultation that an employer would not be able to fulfill. Interestingly, that concern seemed to be a reason employers voiced the greatest support for the third option (services that provide consultation and guidance to both a cancer survivor and their employer), where they could be part of the discussion and involved in identifying reasonable accommodations. Finally, employers worried about the logistics and time demands of peer-based and group support because they might have less flexibility in scheduling group programs around the demands of any given worker’s job and assignments.

## Discussion

### Principal Findings

This study of cancer survivors, health care providers, and employers explored the acceptability of potential interventions to support cancer survivors to continue working during and after cancer treatment. Our goal in this study was not to seek consensus on the exact intervention that should be developed at our cancer center; rather, we sought to gather stakeholder perspectives on approaches to work interventions in the cancer context by highlighting barriers that may need to be addressed as well as identifying both common and unique selling points across varied stakeholders to assist in promoting the uptake of new programs.

These data help to identify the needs and priorities of different stakeholders, many of which have been noted in other studies in the United States and other countries. For example, a qualitative study of employers in Denmark reported inadequate information about how to support cancer survivors at work and emphasized that interventions must also address employers’ needs in tandem with the needs of cancer survivors [23]. Similarly, a US study highlighted employers’ lack of specific knowledge about cancer and the recovery process as a barrier to cancer survivors returning to work [24]. Our findings contribute to a small but growing literature on employment support for cancer survivors in rural environments, where job markets and available resources may differ from those in urban settings [25,26].

Each proposed delivery model has advantages in the eyes of our participants, and whichever intervention we choose to develop will need to be carefully crafted. For example, educational interventions can be helpful but often need to be augmented within a multidisciplinary intervention to be maximally effective [27]. Approaches to increasing rigor in the development of such interventions may include grounding in theory or using frameworks such as the Behavior Change Wheel [28] or the Rehabilitation Treatment Specification System [29] to identify the desired targets and evidence-based behavior change techniques to affect those targets. Principles of effective approaches to improving employment outcomes in other contexts, including job crafting among workers with disabilities [30], cognitive behavioral therapy addressing work challenges among people with mood disorders [31], and individual placement and support for people with serious mental illness and other health conditions [32], may help inform such an intervention.

The feedback gathered in this study can help us to identify implementation strategies that might help us move forward with pilot testing and the development of an employment support program that is feasible and sustainable within our cancer center. The Expert Recommendations for Implementing Change compilation [33] offers a list of implementation strategies that can help systems as they develop or adopt innovations. For example, to maximize the adaptability of any intervention we develop, we could consider having both generic and tailored content available and having rapid tests of change involving “champions” or “early adopters” from a specific demographic who can guide tailoring to their needs (eg, challenges created by certain types of cancer) and environments (eg, types of workplaces). Strategies that can address concerns about compatibility involve finding ways to assess local resources and readiness for change and identify local champions.

### Strengths and Limitations

The 2-stage design of our interview and focus group study allowed for an iterative data collection process along with member checking of key interview findings at the focus group stage. However, a lag of over 6 months between interview completion and focus group scheduling may have contributed to attrition and the small focus group samples, which differed from the interview samples in terms of gender, age, and educational backgrounds. Additionally, these are best described as convenience samples. All 3 participant groups had minimal racial and educational diversity, though cancer survivor demographics were largely consistent with the catchment area of our cancer center. While we oversampled cancer survivors and health care providers, we were unable to acquire our target sample of employers. Few employers responded to our postings, some of them we contacted reported that privacy regulations prevented them from knowing whether any of their employees have had cancer, and others were interested in participating but unavailable at the time of the scheduled focus group sessions.

Regarding the content of the focus groups, we focused on interventions that support survivors remaining in the workforce. We, therefore, omitted suggestions focused on supporting survivors to take extended time off work, providing financial aid, and helping to file disability paperwork. We did not include the perspectives of cancer survivors who left the workforce and may later want to return. We also did not include perspectives from family members of cancer survivors in this study. Furthermore, we decided to map interview participants’ suggestions onto existing intervention delivery models that could be tailored and implemented in a cancer context. The 4 categories of interventions about which we sought feedback were not mutually exclusive; for example, educational approaches could be included in any of the other 3 intervention types. Furthermore, we grouped recommended resources and supports in ways that reflected the number of people involved in their delivery, which may appear superficial or arbitrary; an alternate method of grouping these potential interventions may have produced different results. This design allowed us to gather feedback on a range of interventions from low-touch to high-touch, and to explore pros and cons along this continuum. Any intervention development in this area will benefit from grounding in theory [28] and future work will need to determine which theoretical framing is best suited for informing hypothesized relationships between interventions, mechanisms of action, and employment outcomes for both cancer survivors and employers.

### Conclusions

The variety of intervention advantages and disadvantages identified across participant groups in this study represent potential facilitators and barriers to implementing these types of interventions in practice. Further intervention development, particularly pilot testing stages, would benefit from the application of implementation science perspectives to address these facilitators and barriers.

## Data Availability

The qualitative interview and focus group data collected for this study includes identifiable participant information and is not publicly available. Access to deidentified data may be requested by contacting the corresponding author.
